# Susceptibility to Chronic Mucus Hypersecretion, a Genome Wide Association Study

**DOI:** 10.1371/journal.pone.0091621

**Published:** 2014-04-08

**Authors:** Akkelies E. Dijkstra, Joanna Smolonska, Maarten van den Berge, Ciska Wijmenga, Pieter Zanen, Marjan A. Luinge, Mathieu Platteel, Jan-Willem Lammers, Magnus Dahlback, Kerrie Tosh, Pieter S. Hiemstra, Peter J. Sterk, Avi Spira, Jorgen Vestbo, Borge G. Nordestgaard, Marianne Benn, Sune F. Nielsen, Morten Dahl, W. Monique Verschuren, H. Susan J. Picavet, Henriette A. Smit, Michael Owsijewitsch, Hans U. Kauczor, Harry J. de Koning, Eva Nizankowska-Mogilnicka, Filip Mejza, Pawel Nastalek, Cleo C. van Diemen, Michael H. Cho, Edwin K. Silverman, James D. Crapo, Terri H. Beaty, David A. Lomas, Per Bakke, Amund Gulsvik, Yohan Bossé, M. A. Obeidat, Daan W. Loth, Lies Lahousse, Fernando Rivadeneira, Andre G. Uitterlinden, Andre Hofman, Bruno H. Stricker, Guy G. Brusselle, Cornelia M. van Duijn, Uilke Brouwer, Gerard H. Koppelman, Judith M. Vonk, Martijn C. Nawijn, Harry J. M. Groen, Wim Timens, H. Marike Boezen, Dirkje S. Postma, the LifeLines Cohort study

**Affiliations:** 1 University of Groningen, University Medical Center Groningen, Department of Pulmonology, Groningen, the Netherlands; 2 University of Groningen, University Medical Center Groningen, GRIAC research institute, Groningen, the Netherlands; 3 University of Groningen, University Medical Center Groningen, Department of Genetics, Groningen, the Netherlands; 4 University of Utrecht, University Medical Center Utrecht, Department of Pulmonology, Utrecht, the Netherlands; 5 University of Groningen, University Medical Center Groningen, Department of Pathology and Medical Biology, Groningen, the Netherlands; 6 AstraZeneca, R&D Mölndal, Mölndal, Sweden; 7 AstraZeneca, Alderley Park, Macclesfield, Cheshire, United Kingdom; 8 Department of Pulmonology, Leiden University Medical Center, Leiden, the Netherlands; 9 Department of Respiratory Medicine, University of Amsterdam, Amsterdam, the Netherlands; 10 Department of Medicine, Section of Computational Biomedicine, Boston University Medical Center, Boston, Massachusetts, United States of America; 11 Odense University Hospital, Department of Respiratory Medicine J, and University of Southern Denmark, Clinical Institute, Odense, Denmark; 12 Respiratory and Allergy Research Group, Manchester Academic Health Sciences Centre, University Hospital South Manchester NHS Foundation Trust, Manchester, United Kingdom; 13 The Copenhagen City Heart Study, Frederiksberg Hospital, Copenhagen University Hospital, University of Copenhagen, Copenhagen, Denmark; 14 Department of Clinical Biochemistry, Herlev Hospital, Copenhagen University Hospital, University of Copenhagen, Copenhagen, Denmark; 15 Department of Clinical Biochemistry, Rigshospitalet, Copenhagen University Hospital, University of Copenhagen, Copenhagen, Denmark; 16 Center for Prevention and Health Services Research, National Institute for Public Health and the Environment, Bilthoven, the Netherlands; 17 Julius Center for Health Sciences and Primary Care, UMC Utrecht, Utrecht, the Netherlands; 18 Department of Diagnostic and Interventional Radiology, University Hospital of Heidelberg, Heidelberg, Germany; 19 Translational Lung Research Center (TLRC-H), Member of the German Center for Lung Research (DZL), Heidelberg, Germany; 20 Department of Public Health, Erasmus Medical Center Rotterdam, Rotterdam, the Netherlands; 21 Department of Respiratory Diseases, Jagiellonian University Medical College, Krakow, Poland; 22 Channing Division of Network Medicine, Department of Medicine, Brigham and Women's Hospital, Boston, Massachusetts, United States of America; 23 Division of Pulmonary and Critical Care Medicine, Department of Medicine, Brigham and Women's Hospital, Boston, Massachusetts, United States of America; 24 Harvard Medical School, Boston, Massachusetts, United States of America; 25 Division of Pulmonary and Critical Care Medicine, National Jewish Health, Denver, Colorado, United States of America; 26 Department of Epidemiology, Johns Hopkins Bloomberg School of Public Health, Baltimore, Maryland, United States of America; 27 Cambridge Institute for Medical Research, University of Cambridge, Cambridge, United Kingdom; 28 Department of Thoracic Medicine, Haukeland University, Hospital and Institute of Medicine, University of Bergen, Bergen, Norway; 29 Institut universitaire de cardiologie et de pneumologie de Québec, Department of Molecular Medicine, Laval University, Québec City, Canada; 30 Division of Respirology, Department of Medicine, James Hogg Research Centre, St Paul's Hospital, University of British Columbia, Vancouver, BC, Canada; 31 Dept Epidemiology, Erasmus MC, Rotterdam, the Netherlands; 32 The Netherlands Healthcare Inspectorate, The Hague, the Netherlands; 33 Department of respiratory medicine, University Hospital Ghent, Ghent, Belgium; 34 Department of Internal medicine, Erasmus MC, Rotterdam, the Netherlands; 35 Netherlands Consortium for Healthy Aging (NCHA), Rotterdam, the Netherlands; 36 Genetic Epidemiology Unit, Department of Epidemiology, Erasmus Medical Center, University Medical Center, Rotterdam, the Netherlands; 37 University of Groningen, University Medical Center Groningen, Beatrix Childrens' Hospital, Department of Pediatric Pulmonology and Pediatric Allergology, Groningen, the Netherlands; 38 University of Groningen, University Medical Center Groningen, Department of Epidemiology, Groningen, the Netherlands; University of Tübingen, Germany

## Abstract

**Background:**

Chronic mucus hypersecretion (CMH) is associated with an increased frequency of respiratory infections, excess lung function decline, and increased hospitalisation and mortality rates in the general population. It is associated with smoking, but it is unknown why only a minority of smokers develops CMH. A plausible explanation for this phenomenon is a predisposing genetic constitution. Therefore, we performed a genome wide association (GWA) study of CMH in Caucasian populations.

**Methods:**

GWA analysis was performed in the NELSON-study using the Illumina 610 array, followed by replication and meta-analysis in 11 additional cohorts. In total 2,704 subjects with, and 7,624 subjects without CMH were included, all current or former heavy smokers (≥20 pack-years). Additional studies were performed to test the functional relevance of the most significant single nucleotide polymorphism (SNP).

**Results:**

A strong association with CMH, consistent across all cohorts, was observed with rs6577641 (p = 4.25×10^−6^, OR = 1.17), located in intron 9 of the *special AT-rich sequence-binding protein 1 locus* (*SATB1*) on chromosome 3. The risk allele (G) was associated with higher mRNA expression of *SATB1* (4.3×10^−9^) in lung tissue. Presence of CMH was associated with increased *SATB1* mRNA expression in bronchial biopsies from COPD patients. *SATB1* expression was induced during differentiation of primary human bronchial epithelial cells in culture.

**Conclusions:**

Our findings, that SNP rs6577641 is associated with CMH in multiple cohorts and is a *cis*-eQTL for *SATB1*, together with our additional observation that *SATB1* expression increases during epithelial differentiation provide suggestive evidence that *SATB1* is a gene that affects CMH.

## Introduction

The secretion of mucus is a natural part of the airway defense against inhaled noxious particles and substances. Chronic mucus hypersecretion (CMH) is a condition of overproduction of mucus and defined as the presence of sputum production during at least three months in two consecutive years without any explaining origin whereas airway obstruction is not a prerequisite [1]. Smoking is a risk factor for CMH, i.e. the prevalence of CMH in the general population is reported to be 7.4% in current smokers, 3.7% in ex-smokers and 2.4% in never smokers [2]. CMH is the key presenting symptom in chronic bronchitis, one of the three main sub-groups of chronic obstructive pulmonary disease (COPD), a complex disease characterized by the presence of incompletely reversible and generally progressive airflow limitation [3]. Moreover, CMH is a risk factor for the development of COPD [4], [5].

Worldwide, COPD affected 65 million people in 2004 and more than 3 million people died of COPD in 2005, representing 5% of all deaths. It is predicted that COPD will be the third leading cause of death worldwide in 2030 [6]. COPD markedly reduces quality of life and is responsible for high healthcare costs. For instance, the combined (direct and indirect) yearly costs of COPD and asthma in the United States of America were projected at $68 billion in 2008 [7]. CMH is not only associated with COPD but also with an increased duration and frequency of respiratory infections, excess decline in forced expiratory volume in 1 second (FEV_1_) and increased hospitalization and mortality rates in the general population [4], [5], [8], [9].

It is not known why only a minority of all smokers develops CMH, yet a plausible explanation is the presence of a genetic predisposition for CMH, as evidenced by familial aggregation of mucus overproduction and higher prevalence of CMH in monozygotic than in dizygotic twins [10]–[12]. Little is known about the identity of the genes that predispose to CMH. One publication suggested that *CTLA4* is associated with chronic bronchitis in COPD [13].

The aim of our study was to identify genetic factors for CMH, thereby obtaining a better insight into the origins of this disorder.

## Materials and Methods

### Ethics Statement

The Dutch ministry of health and the Medical Ethics Committee of the hospital approved the study protocol for all Dutch centers. Ethics approval and written informed consent was obtained from all participants in all studies participating. For detailed information, see Supplement S1.

### Subjects and genotyping

We performed GWA studies in participants of the NELSON-study (n = 3,729), a male population-based lung cancer screening study investigating heavy smokers (≥20 pack-years) [24].

Replication of SNPs with p≤10^−4^ was attempted in six cohorts participating in ‘COPD Pathology: Addressing Critical gaps, Early Treatment & diagnosis and Innovative Concepts’ (COPACETIC) and in five non-COPACETIC cohorts. Caucasian subjects with ≥20 pack-years smoking with genotype-, spirometric- and demographic data were included.

An overview of the CMH definitions used in this study is presented in Table 1. A brief description of the included cohorts and details according to the period of data collection, type of population, genotyping platforms and genetic imputation software are presented in in Table 2.

**Table 1:** Questions used to define chronic mucus hypersecretion in the corresponding cohorts.

Cohort	Question
NELSON [24]	Do you expectorate sputum on the majority of days more than 3 months a year, even when you do not have a cold?
Rotterdam [25], [26]	Do you expectorate sputum on the majority of days during ≥3 months during the last 2 years?
LifeLines [27]	Do you usually expectorate sputum during day or night in winter? If yes: Do you expectorate sputum on the majority of days >3 months a year?
Vlagtwedde- Vlaardingen [28], [29]	Do you expectorate sputum on the majority of days >3 months a year?
Doetinchem [30]	Do you expectorate sputum during winter, day and night, each day for 3 months?
Poland [31], [32]	Do you usually bring up phlegm from your chest, or do you usually have phlegm in your chest that is difficult to bring up when you don't have a cold? If yes: Are there months in which you have this phlegm on most days? If yes: Do you bring up this phlegm on most days for as much as three months each year? A positive answer to all (3) questions identifies CMH.
Heidelberg [33]	Do you expectorate sputum on the majority of days >3 months a year?
GLUCOLD [15]	Do you expectorate sputum immediately after getting up on the majority of days in winter >3 months a year?
Rucphen [29]	Do you expectorate sputum during day or night in winter? If yes: Do you have expectoration on the majority of days >3 months a year?
ECLIPSE [34]	Do you usually bring up phlegm from your chest on getting up, first thing in the morning, during the rest of the day or at night, on most days for 3 consecutive months or more during the year?
COPDGene [35]	Do you usually bring up phlegm from your chest on getting up, first thing in the morning, during the rest of the day or at night, on most days for 3 consecutive months or more during the year?
Norway [36], [37]	Do you usually bring up phlegm from your chest on getting up, first thing in the morning, during the rest of the day or at night, on most days for 3 consecutive months or more during the year?

**Table 2:** Overview of populations.

Study	Data Collection	Type of population	Genotyping platform	Imputation software
NELSON	2004/2005	general population	Illumina Quad 610	NA
GLUCOLD	2005/2006	COPD case	Illumina Veracode	NA
Vlagtwedde Vlaardingen	1989/1990	general population	Illumina Veracode	NA
Doetinchem	1998/2002	general population	Illumina Veracode	NA
Poland	2005/2006	general population	Illumina Veracode	NA
Heidelberg	2004/2005	general population	Illumina Veracode	NA
Rucphen	2002	Family based COPD on a doctor diagnosis	Illumina Veracode	NA
Rotterdam	2002/2008	general population	Illumina 550K	MaCH
LifeLines	2008/2010	general population	Illumina Human CytoSNP-12	BEAGLE v3.1.0
COPDGene	2008/2009	COPD case/control (stage I–IV)	Illumina Human Omni1-Quad	MaCH
ECLIPSE	2005/2007	COPD case/control (stage II–IV)	Illumina Human HAP 550 V3	MaCH
Norway	2003/2005	COPD case/control (stage II–IV)	Illumina Human HAP 550 V1, V3, and DUO	MaCH

Populations and corresponding period of data collection, type of population, genotyping platform and soft-ware used for imputation.

NA = not applicable.

### Strategy

**Figure 1:**
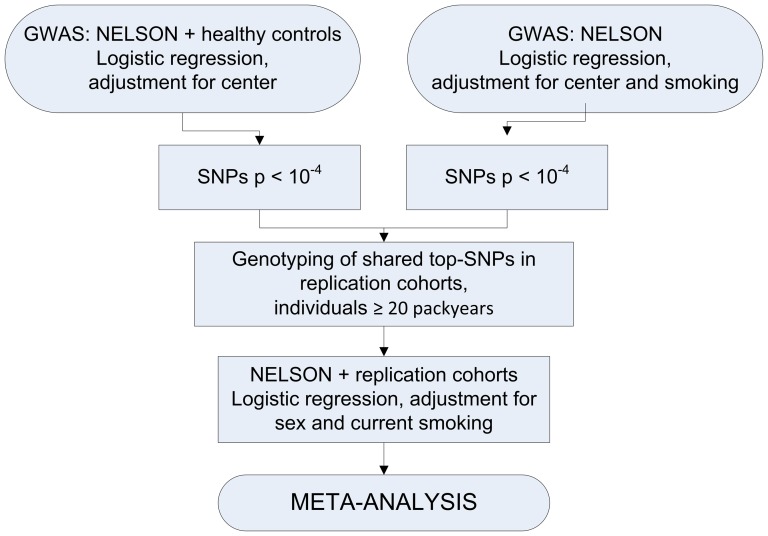
Study design. We performed GWA studies in the NELSON cohort and in additional healthy controls. CMH was analyzed using logistic regression with adjustment for center (Groningen and Utrecht). Since current smoking can affect the presence of CMH, we additionally performed the GWAS in the NELSON cohort correcting for center and smoking. SNPs with a p-value<10-4 present in both GWA studies were selected for replication. To test for generalizability of associations with CMH in other populations, we compared our results with data in CMH-cases and controls with a smoking history of ≥20 pack-years with eleven replication populations using logistic regression with adjustment for sex and current smoking. Finally, we performed a meta-analysis on shared SNPs across the NELSON identification population and the 11 replication populations.

We searched for SNPs associated with CMH by using a two-stage strategy followed by a replication stage and meta-analysis (Figure 1).

### Statistical analysis

General characteristics of CMH-cases and controls were compared using Student's t- and Mann-Whitney-U tests for continuous variables and χ2 tests for dichotomous variables with SPSS 20.0. Sample and SNP quality control (QC), regression- and meta-analysis were performed with PLINK 1.07 [25]. QC criteria are described in Supplement S1.

Logistic regression analysis under an additive model was used to identify SNPs associated with CMH. SNPs with a p-value<10^−4^ were included for replication. When two SNPs were in strong linkage disequilibrium (r2≥0.8), the SNP with the lowest p-value was further analyzed.

SNPs in COPACETIC cohorts and in LifeLines were analyzed using logistic regression with adjustment for sex and smoking (ex-/current smoking). In LifeLines, imputed SNPs with an info-score <0.3 (imputation quality score) were removed. SNPs in non-COPACETIC cohorts were analyzed by the cohort investigators using the same model.

Meta-analysis was performed on SNPs across NELSON and the 11 replication cohorts. The Cochran's Q test was used to test for heterogeneity in the meta-analysis.

We performed multivariate logistic regression analysis, adjusted for pack-years and lung function, to associate CMH with the risk allele of rs6577641 in the identification cohort.

### Functional relevance of SATB1 and rs6577641, our highest ranked-SNP

We performed 4 functional studies with the identified top-SNP. Details on their methods are given in Supplement S1.

We assessed:

1. whether rs6577641 is an eQTL, by analyzing the association of SATB1 expression levels with rs6577641 genotypes in lung tissue from three independent cohorts recruited from Laval University, University of British Columbia, and University of Groningen as described previously [14];
2. CMH-associated mRNA expression in airway wall biopsies from 77 COPD participants in the GLUCOLD-study [15];
3. the association of homozygous genotypes for rs6577641 with a) immunohistochemical staining (IHC) for SATB1 and b) the fraction of mucus positivity on bronchial tissue explanted from COPD or lung cancer subjects that underwent lung surgery;
4. SATB1 expression levels during mucociliary differentiation of primary bronchial epithelial cells cultured at air-liquid interface [26].

## Results

### Populations

Characteristics of the identification and replication populations are presented in Table 3. Subjects with CMH were more often current smokers and had worse lung function, except for populations including subjects with COPD only.

**Table 3:** Demographic and clinical characteristics of CMH-cases and -controls with ≥20 pack-years, present in the meta-analysis.

Population	CMH	N	Population %	Female, %	Age, yrs (SD)	Pack-years (range)	Current smoking, %	FEV1 %, pred. (SD)	FEV1/FVC, % (SD)
NELSON	Control	1,795	71.5	0	60.2 (5.3)	34 (21–156)	47.5	100.3 (17.2)	72.9 (8.7)
NELSON	Case	717	28.5	0	60.4 (5.6)	39 (21–140)	74.2	93.5 (20.0)	69.2 (11.0)
Rotterdam	Control	1,043	84.1	46.1	68.0 (9.3)	45 (20–149)	40.1	92.4 (23.5)#	72.8 (8.7)#
Rotterdam	Case	197	15.9	43.7	72.0 (8.4)	40 (20–168)	45.2	85.0 (26.9)#	68.0 (11.1)#
LifeLines	Control	1,431	88.1	80.1	52.9 (9.2)	27 (20–100)	56.4	98.2 (15.6)	72.4 (8.2)
LifeLines	Case	193	11.5	46.9	53.2 (9.9)	29 (20–97)	75.4	90.5 (18.0)	68.3 (11.3)
Vlagtwedde-Vlaardingen*	Control	234	82.4	27.4	52.9 (10.1)	29 (20–128)	51.7	94.5 (12.1)	76.6 (4.5)
Vlagtwedde-Vlaardingen*	Case	50	17.6	18	53.4 (10.5)	33 (22–83)	68	86.7 (18.6)	71.0 (8.9)
Doetinchem	Control	250	80.6	37.2	54.7 (8.8)	30 (20–90)	55.6	94.8 (17.6)	71.5 (9.9)
Doetinchem	Case	60	19.4	36.7	56.4 (7.7)	33 (20–72)	68.3	89.1 (19.6)	69.3 (11.4)
Poland	Control	97	85.1	22.7	56.7 (10.5)	30 (20–116)	52.6	96.4 (21.4)	72.5 (0.5)
Poland	Case	17	14.9	11.8	55.8 (9.4)	35 (22–86)	82.4	93.5 (24.0)	69.2 (13.1)
Heidelberg	Control	608	84.2	35.7	58.1 (5.2)	37 (23–138)	54.3	96.4 (17.6)	78.9 (9.7)
Heidelberg	Case	114	15.8	29.8	58.0 (5.2)	37 (23–91)	91.2	86.2 (21.5)	75.3 (10.6)
GLUCOLD**	Control	48	55.2	8.3	62.6 (7.6)	46 (21–182)	62.5	63.4 (9.8)	50.4 (9.1)
GLUCOLD**	Case	39	44.8	20.5	59.6 (7.4)	40 (22–83)	61.5	63.9 (8.8)	53.1 (7.8)
Rucphen**	Control	28	53.8	46.4	66.5 (7.9)	42 (21–120)	57.1	74.5 (15.7)	57.2 (7.8)
Rucphen**	Case	24	46.2	41.7	62.2 (10.5)	43 (21–100)	70.8	70.2 (21.6)	53.1 (9.7)
ECLIPSE**	Control	961	62	37.5	64.1 (6.7)	53 (21–205)	28.1	48.0 (15.7)	44.5 (11.3)
ECLIPSE**	Case	590	38	24.1	62.9 (7.4)	54 (22–220)	47.5	46.2 (15.5)	44.3 (11.7)
COPDGene	Control	628	71.8	53.5	63.1 (8.6)	50 (21–173)	28.2	75.0 (28.3)	63.7 (17.6)
COPDGene	Case	247	28.2	40.5	61.9 (8.4)	54 (21–237)	50.2	60.4 (27.4)	54.6 (17.9)
Norway	Control	501	52.4	44.9	61.5 (10.3)	34 (20–130)	46.9	71.7 (24.2)	64.6 (15.7)
Norway	Case	456	47.6	20.4	64.1 (10.1)	39 (20–119)	59	56.5 (24.4)	55.0 (17.3)

CMH = Chronic mucus hypersecretion;

*lung function is based on FEV_1_/IVC;

**all individuals in this cohort have COPD;

# based on lung function of 700 subjects who returned for follow-up study 4 years later.

### Identification analysis

**Figure 2:**
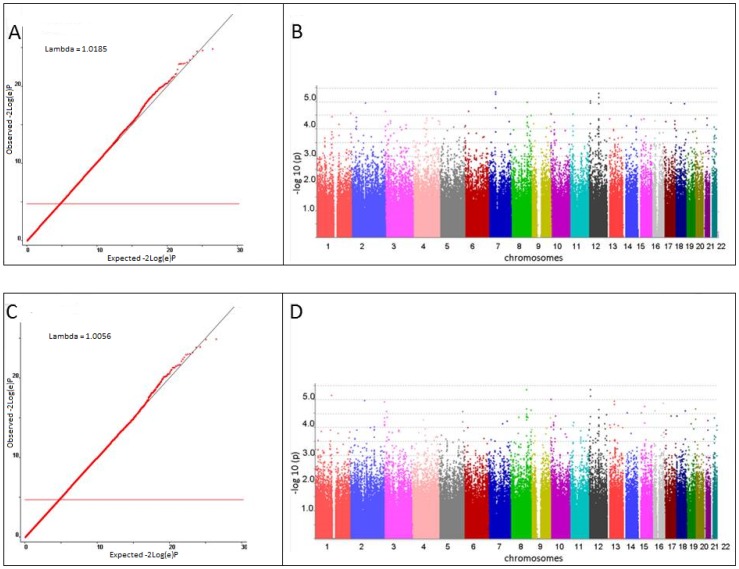
Quantile-quantile plot and Manhattan plot of GWA results for association of SNPs with CMH in NELSON amplified with bloodbank controls and corrected for center (A and B). Quantile-quantile plot and Manhattan plot of GWA results for association of SNPs with CMH in NELSON, corrected for center and smoking habits (C and D).

After QC, 492,700 SNPs and 2,512 individuals (717 CMH cases, 1,795 controls) from the NELSON study remained. Logistic regression analysis was performed including these individuals supplemented with 590 additional healthy controls, adjusting for center. The QQ-plot provided no evidence of population stratification (λ = 1.0185). 77 SNPs were associated with CMH with a p-value<10^−4^. CMH was associated with current smoking in our identification cohort (p<0.001). Therefore, we performed a second GWA adjusting for center and current/ex-smoking (717 CMH-cases, 1,795 controls). The QQ-plot showed no evidence of population stratification (λ = 1.0056). We observed 64 SNPs with a p-value<10^−4^. Genome wide association for CMH ordered by chromosome is shown in the Manhattan plot. Figure 2 shows QQ-plots (A, C) and genome wide association signals for CMH ordered by chromosome (Manhattan-plots, B and D) of these sequential analyses. We identified 36 SNPs associated with CMH with a p-value<10^−4^ in both analyses Table 4. Of these, 32 SNPs were included for replication and 4 SNPs were removed because they were in strong linkage disequilibrium (r^2^>0.8) with another associated SNP.

**Table 4:** SNPs associated with CMH with a p-value<10^−4^, present in GWAS-I and in GWAS-II, in the NELSON identification cohort.

Chromosome	SNP	Base pair position	p-value GWAS I	p-value GWAS II
2	rs6735868	103582093	1.11×10^−05^	1.08×10^−05^
3	rs1387089	1940922	7.94×10^−05^	4.56×10^−05^
3	rs1488757	1981567	2.17×10^−05^	1.16×10^−05^
3	rs6577641	18397849	6.83×10^−05^	2.57×10^−05^
4	rs4306981	79924121	9.74×10^−05^	5.18×10^−05^
8	rs4242562	115475287	7.66×10^−05^	5.13×10^−05^
8	rs7836298	115504434	1.03×10^−05^	4.37×10^−06^
8	rs7823554*	115553109	6.05×10^−05^	5.22×10^−05^
8	rs7836963*	115568426	5.52×10^−05^	4.24×10^−05^
8	rs16886291	115711436	3.54×10^−05^	2.09×10^−05^
8	rs10098746	125838127	8.47×10^−05^	4.34×10^−05^
8	rs7831595	144974963	3.08×10^−05^	2.32×10^−05^
9	rs4842047	138816796	2.63×10^−05^	4.51×10^−05^
10	rs943189	22842590	5.57×10^−05^	6.33×10^−05^
11	rs11026531	22379184	2.76×10^−05^	8.55×10^−05^
12	rs1894307*	12005720	9.04×10^−06^	7.18×10^−06^
12	rs2255953	12010736	1.13×10^−05^	4.33×10^−06^
12	rs2855708	12013572	6.47×10^−05^	3.97×10^−05^
12	rs10879509*	73242131	6.98×10^−06^	4.44×10^−05^
12	rs4760851	73284781	4.85×10^−06^	2.29×10^−05^
12	rs952394	73441110	4.18×10^−05^	4.22×10^−05^
12	rs12822199	75458164	4.82×10^−05^	8.58×10^−05^
12	rs1379963	75493882	1.18×10^−05^	2.20×10^−05^
12	rs1795669	76273692	8.01×10^−05^	7.86×10^−05^
13	rs9578362	21882381	4.28×10^−05^	7.99×10^−05^
13	rs1211304	50381016	9.96×10^−05^	1.12×10^−05^
14	rs992745	27810095	7.67×10^−05^	2.99×10^−05^
15	rs754661	26934277	4.54×10^−05^	2.88×10^−05^
15	rs4775569	46850317	4.20×10^−05^	1.72×10^−05^
16	rs13333521	19904082	5.08×10^−05^	2.50×10^−05^
17	rs11652469	49565797	1.13×10^−05^	3.80×10^−05^
18	rs8086262	69227590	1.15×10^−05^	2.53×10^−05^
20	rs4815628	3891896	4.17×10^−05^	2.15×10^−05^
21	rs2032257	27774870	3.97×10^−05^	5.39×10^−05^
22	rs1009147	30088257	8.41×10^−05^	4.51×10^−05^
22	rs1005239	47687170	9.86×10^−05^	8.67×10^−05^

*SNP not selected for replication because of strong linkage disequilibrium with another SNP.

### Replication of associated SNPs

Genotyping of SNP rs4775569 failed in the COPACETIC populations, and was removed for further analysis. CMH-associated top-SNPs for each cohort are presented in Table 5, with a complete overview in Table 6. When applying Bonferroni correction in the meta-analysis (p = 1.61×10^−3^ for 31 SNPs), we found a strong association with one SNP:

**Table 5:** Meta-analysis of top SNPs associated with CMH in replication cohorts, in identification and replication cohorts and corresponding direction of effect in all cohorts and associated feature and gene(s).

					Meta-analysis across replication cohorts		Meta-analysis across identification and replication cohorts				
Chr	SNP	Base pair position	Minor allele	MAF	p-value	OR	Direction of effect per cohort*	p-value	OR	Q	Close(st) gene(s
3	rs6577641	18397849	G	0.400	5.01E-03	1.12	++++++++0+0+	4.25 E-06	1.17	6.20E-01	*SATB1* #
3	rs1488757	1981567	G	0.109	2.34E-01	0.92	-00+--+++--0	1.10E-03	0.83	1.55E-01	*LOC727810 and CNTN4*
12	rs2855708	12013572	G	0.273	2.18E-01	1.06	+0+0--+-+++0	1.20E-03	1.13	1.76E-01	*ETV6* #
14	rs992745	27810095	G	0.234	2.94E-01	0.95	--+----+++--	2.74E-03	0.89	4.59E-02	*LOC7288755* #
4	rs4306981	79924121	G	0.307	3.37E-01	1.04	++-0-+++-0-+	1.38E-03	1.12	5.19E-02	*PAQR3 and ARD1B*
12	rs1795669	76273692	A	0.059	2.83E-01	1.09	+++++----+++	2.90E-03	1.22	1.77E-01	*LOC100130336 and LOC100131830*
9	rs4842047	137956617	A	0.303	3.88E-01	0.96	-0-XX+-X0-00	3.44E-03	0.89	3.03E-01	*CAMPSAP1 and UBAC1*
13	rs95788362	21882381	A	0.402	8.05E-01	1.01	-+---+0+--00	3.61E-03	0.91	2.88E-02	*LOC6500794 and GRK6PS*
12	rs2255953	12010736	G	0.212	5.31E-01	0.97	+-X---+-0++0	5.12E-03	1.13	4.54E-02	*ETV6* #
15	rs754661	26934277	G	0.405	5.45E-01	0.96	-00X-++---0+	6.29E-03	0.91	1.08E-01	*GABRB3* #
8	rs16886291	115711436	A	0.127	5.01E-03	1.12	---+--+-+00+	5.41E-03	0.86	1.55E-01	*hCG_1644355 and TRPS1*

MAF = minor allele frequency in NELSON;

*Direction of effect per cohort: each sign reflects one cohort, direction of effect is presented by: + = (OR>1.05), − = (OR<0.95), 0 = (0.95<OR<1.05) and x = (missing result); cohorts are presented in the same order as in Table 2; OR is odds ratio; Q is p-value for heterogeneity;

# means corresponding SNP is located in an intron in this gene.

**Table 6:** Meta-analysis of top SNPs associated with CMH across replication cohorts and across identification and replication cohorts, corrected for smoking and sex.

				Meta-analysis across replication cohorts			Meta-analysis across identification and replication cohorts				
Chromosome	Base pair position	SNP	Minor allele	p-value	OR	Q	N	p-value	OR	Q	Close(st) gene(s)
3	18397849	rs6577641	G	5.01E-03	1.121	9.19E-01	12	4.25E-06	1.173	6.20E-01	*SATB1* *
18	69227590	rs8086262	G	2.16E-02	1.107	1.00E-02	11	8.91E-02	1.129	2.60E-03	*LOC100132647 and CBLN2*
8	115475287	rs4242562	C	5.04E-02	1.175	4.20E-01	12	6.15E-01	1.073	1.40E-03	*hCG_1644355 and TRPS1*
8	125838127	rs10098746	A	5.74E-02	0.906	1.76E-01	9	6.22E-01	0.954	2.00E-04	*MTSS1 and LOC100130448*
12	75493882	rs1379963	A	8.87E-02	1.089	6.08E-01	11	4.57E-01	1.063	2.10E-03	*KCNC2* *
12	75458164	rs12822199	G	9.66E-02	1.093	5.30E-01	12	8.50E-01	1.016	4.70E-03	*KCNC2* *
13	50381016	rs1211304	A	1.37E-01	1.099	6.86E-01	11	8.70E-01	0.983	2.50E-03	*KPNA3 and LOC220429*
12	12013572	rs2855708	G	2.18E-01	1.057	7.02E-01	12	1.20E-03	1.132	1.76E-01	*ETV6* *
3	1981567	rs1488757	G	2.34E-01	0.923	8.44E-01	12	1.10E-03	0.828	1.55E-01	*LOC727810 and CNTN4*
12	76273692	rs1795669	A	2.83E-01	1.087	7.02E-01	12	2.90E-03	1.218	1.77E-01	*LOC100130336 and LOC100131830*
14	27810095	rs992745	G	2.94E-01	0.952	4.06E-01	12	2.74E-03	0.886	4.59E-02	*LOC728755* *
13	21882381	rs9578362	A	3.06E-01	0.96	2.06E-01	12	3.61E-03	0.905	2.88E-02	*LOC650794 and GRK6PS*
4	79924121	rs4306981	G	3.37E-01	1.043	3.45E-01	12	2.89E-03	1.117	5.19E-02	*PAQR3 and ARD1B*
2	103582093	rs6735868	A	3.66E-01	1.051	8.51E-01	12	1.59E-01	0.936	1.38E-02	*TMEM182 and LOC728815*
9	137956617	rs4842047	A	3.88E-01	0.961	9.99E-01	9	3.44E-03	0.893	3.03E-01	*CAMSAP1 and UBAC1*
17	49565797	rs11652469	G	4.51E-01	0.942	2.39E-01	12	9.77E-01	1.004	3.50E-03	*FLJ42842 and LOC388401*
15	26934277	rs754661	G	5.31E-01	0.974	8.08E-01	11	6.29E-03	0.908	1.08E-01	*GABRB3* *
8	115711436	rs16886291	A	5.45E-01	0.963	8.77E-01	12	6.47E-03	0.864	1.32E-01	*hCG_1644355 and TRPS1*
20	3891896	rs4815628	A	5.90E-01	1.022	2.76E-01	12	4.51E-01	0.954	3.90E-03	*PANK2* *
10	22842590	rs943189	A	6.12E-01	1.022	6.86E-01	12	8.82E-02	0.94	3.59E-02	*SPAG6 and LOC643475*
12	73284781	rs4760851	A	6.15E-01	1.021	9.57E-01	12	7.51E-02	0.94	6.50E-02	*TRHDE and LOC100128674*
3	1940922	rs1387089	G	6.80E-01	0.972	4.43E-01	11	1.13E-02	0.861	3.64E-02	*LOC391504 and LOC727810*
22	47687170	rs1005239	G	7.07E-01	0.984	5.10E-01	12	1.44E-02	0.918	6.50E-02	*TBC1D22A and RP11-191L9.1*
21	27774870	rs2032257	A	7.14E-01	1.015	3.24E-01	12	7.68E-02	0.94	9.90E-03	*APP and CYYR1*
8	115504434	rs7836298	G	7.16E-01	1.026	6.19E-01	12	5.60E-02	0.889	7.20E-03	*hCG_1644355 and TRPS1*
12	73441110	rs952394	G	7.31E-01	0.986	9.15E-01	12	5.54E-02	1.068	8.47E-02	*TRHDE and LOC100128674*
12	12010736	rs2255953	G	8.05E-01	1.013	8.06E-01	11	5.12E-03	1.131	4.54E-02	*ETV6* *
16	19904082	rs13333521	A	8.34E-01	1.022	6.71E-02	9	3.11E-01	1.181	2.70E-03	*GPRC5B and GPR139*
22	30088257	rs1009147	A	8.58E-01	0.989	9.91E-01	11	2.07E-02	0.881	2.06E-01	*NF2* *
8	144974963	rs7831595	A	8.96E-01	1.005	1.82E-01	12	1.90E-02	1.084	5.90E-03	*EPPK1*
11	22379184	rs11026531	A	9.75E-01	1.002	3.72E-01	11	3.49E-02	0.916	2.31E-02	*SLC17A6* *

P-value is fixed p-value if p-value for heterogeneity (Q) >0.005, and random p-value if p-value for heterogeneity (Q) <0.005; OR is Odds Ratio; OR is fixed OR if p-value for heterogeneity (Q) >0.005, and random OR if p-value for heterogeneity (Q) <0.005; Q is p-value for heterogeneity;

N = number of cohorts;

*means that the corresponding SNP is an intron in this gene.

- rs6577641, a SNP located on chromosome 3 in intron 9 of the *special AT-rich sequence-binding protein 1 locus* (*SATB1*) (combined p-value = 4.25×10^−6^, OR = 1.17; 1.10–1.26).

**Figure 3:**
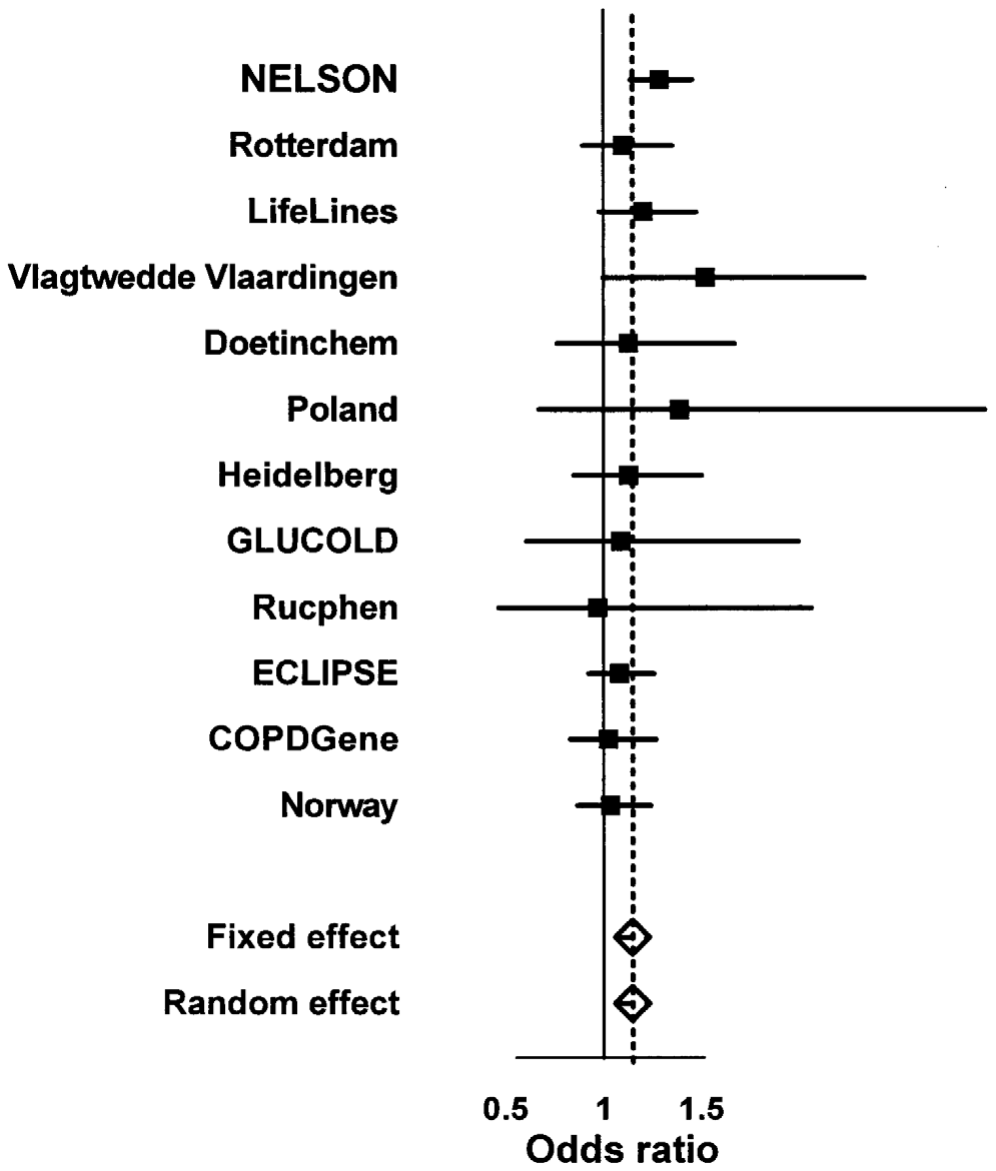
Forest plot showing evidence of association for rs6577641 with chronic mucus hypersecretion in the identification and replication cohorts. Vertically left, the identification cohort and the replication cohorts included in the meta-analysis. The boxes represent the precision and the horizontal lines represent the confidence intervals. The squares represent the pooled effect estimate from the meta-analysis of all cohorts. The horizontal axis shows the scale of the effects.

The *SATB1* SNP rs6577641 had the lowest p-value for association with CMH in the meta-analysis. Figure 3 shows the forest plot of rs6577641 in the identification and replication cohorts and meta-analysis.

**Figure 4:**
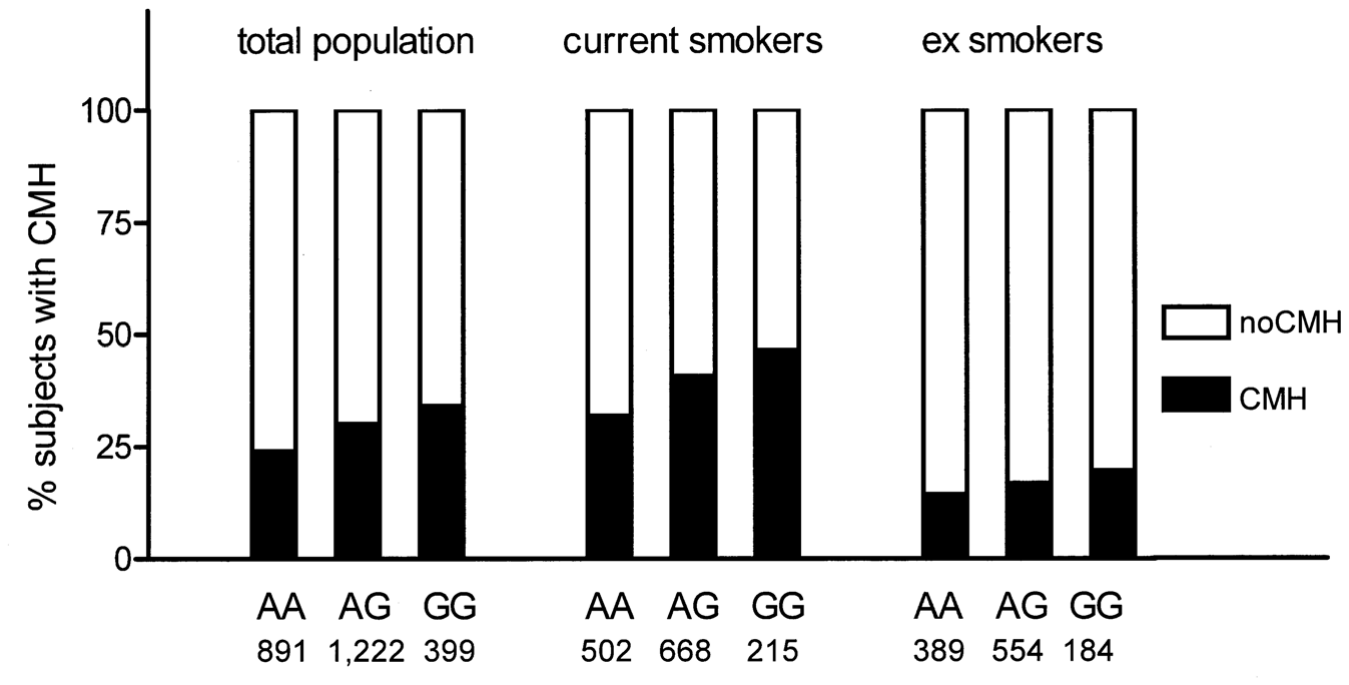
Percentage of subjects with chronic mucus hypersecretion (CMH) within genotypes (AA, AG and GG) of rs6577641 in the identification cohort (NELSON), and distributed among ex- and current smokers.

We assessed the percentage of subjects with CMH in each genotyping group for rs6577641 in NELSON-total and stratified for current and ex smokers (Figure 4). Multivariate logistic regression analysis, corrected for pack-years and FEV_1_%predicted, showed that CMH was significantly associated with the number of G-alleles in the 1,385 current smokers (reference = AA: heterozygous mutant (AG) p = 0.001; OR = 1.50, homozygous mutant (GG) p = 0.001; OR = 1.80) but not in 1,127 ex-smokers (reference = AA: heterozygous mutant (AG) p = 0.380; OR = 1.18, homozygous mutant (GG) p = 0.143; OR = 1.42).

### Functional relevance of SATB1 and rs6577641

1) Transcriptional regulation of *SATB1* mRNA expression

We analyzed the association of *SATB1* expression levels in lung tissue with rs6577641 genotype in 3 independent data sets of the Universities of Groningen, Laval and UBC [14]. A *cis*-acting effect of rs6577641 on *SATB1* expression was identified and present in all three datasets (n = 1,095), with the same direction of effect across all three *SATB1* probes on the array. The (susceptibility) G allele increased expression, the (protective) A allele reduced expression (p = 4.3×10^−9^) in the meta-analysis across the three datasets and across all three SATB1 probes measured (Table 7).

**Table 7:** Meta-analysis of the effect of rs6577641 on mRNA expression levels of *SATB1* in the lung*.

Probe Gene Symbol	Affymetrix Probe ID	Z-score Groningen	Z-score Laval	Z-score UBC	Z-Score Meta-Analysis	p-value Meta-Analysis
		N = 351	N = 335	N = 409		
*SATB1*	100148784_TGI_at	−2.28	−0.08	−1.62	−2.29	**0.022**
*SATB1*	100150253_TGI_at	−0.84	−0.49	−1.62	−1.70	0.088
*SATB1*	100305926_TGI_at	−2.81	−1.38	−1.46	−3.26	**0.001**

*To assess the effect of the SNP rs6577641 on gene expression, a Kruskal-Wallis test was performed. This test generates a p-value, but does not give a direction of the effect. To assess the direction of the effect, a Spearman's correlation test was performed. Next, a Z-score was calculated for each center and a meta-analysis performed for each of the three *SATB1* probes across all centers. Finally, a meta-analysis for all three *SATB1* probes was performed across all centers. This generated a Z-score of −5.87 and a corresponding p-value of 4.3*10^−9^, indicating that the susceptibility G allele of the SNP rs6577641 increases *SATB1* expression.

2) *SATB1* mRNA expression and CMH

**Figure 5:**
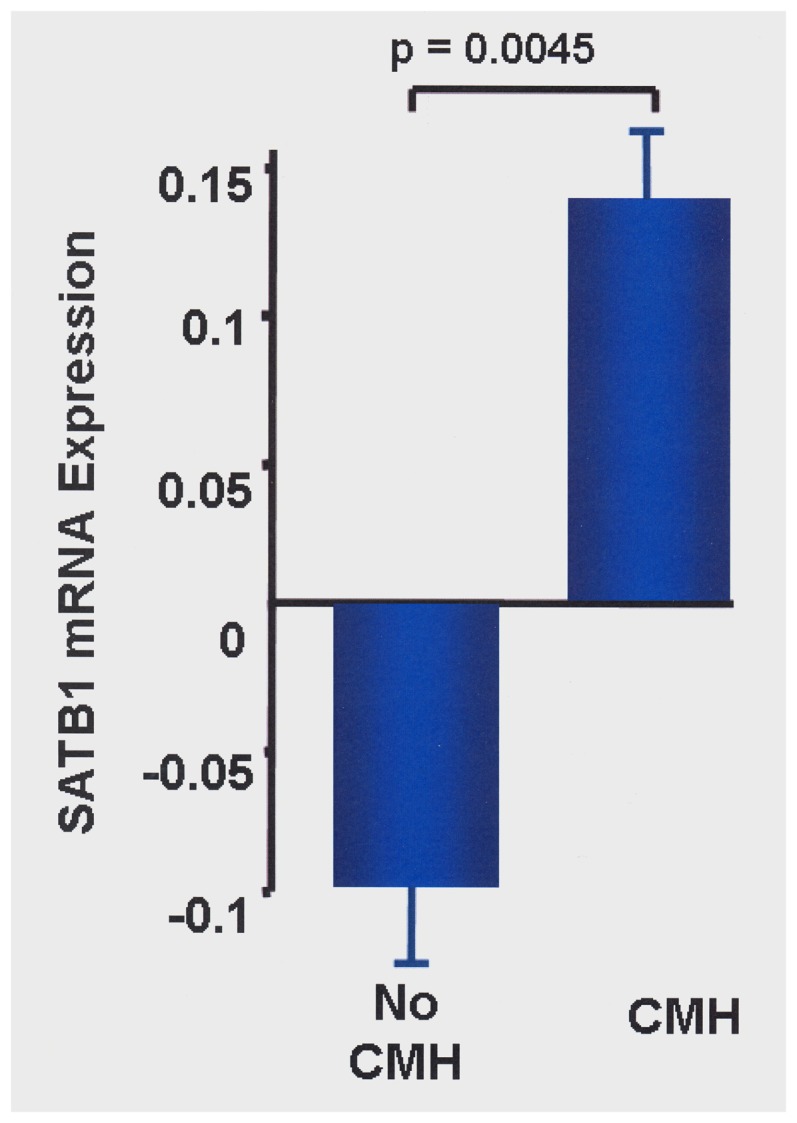
Bronchial biopsy mRNA-expression levels of SATB1 in COPD patients with chronic mucus hypersecretion (n = 38) compared to patients without chronic mucus hypersecretion (n = 39).

We compared *SATB1* expression in baseline airway wall biopsies of COPD patients with (n = 38) and without (n = 39) CMH in GLUCOLD [15]. CMH was significantly associated with *SATB1* expression levels (corrected for ex-/current smoking; p = 0.0045; Figure 5). After stratification, the same direction of effect was present in ex- and current smokers. However, this association reached statistical significance in current smokers (p = 0.021) and not in ex- smokers (p = 0.132), probably due to a difference in power as 46 subjects were current smokers versus 33 ex-smokers.

3) Genotype related protein expression and mucus positivity in bronchial epithelium

SATB1 protein expression has previously been observed in IHS analysis of bronchial epithelial cells [16]. Therefore, we stained SATB1 on paraffin embedded lung tissue biopsies of individuals from the Groningen population contributing to the eQTL analysis. We observed clear nuclear staining for SATB1 in bronchial epithelial cells. No significant difference for % of strong positive, positive and weak positive cells was observed between the protective (AA, n = 9) and risk (GG, n = 14) rs6577641 genotypes (11.8%±5.8 versus 12.7%±6.9, p = 0.74).

We determined whether the fraction of mucus positive bronchial epithelium was different in subjects with different homozygous rs6577641 genotypes and performed PAS-staining on tissue biopsies from the same cohort. We observed no significant difference between individuals with the homozygous protective (AA, n = 10) and risk (GG, n = 7) alleles (19.7%±11.9 versus 14.3%±9.6, p = 0.34).

4) *SATB1* expression levels during bronchial epithelial cell mucociliary differentiation

**Figure 6:**
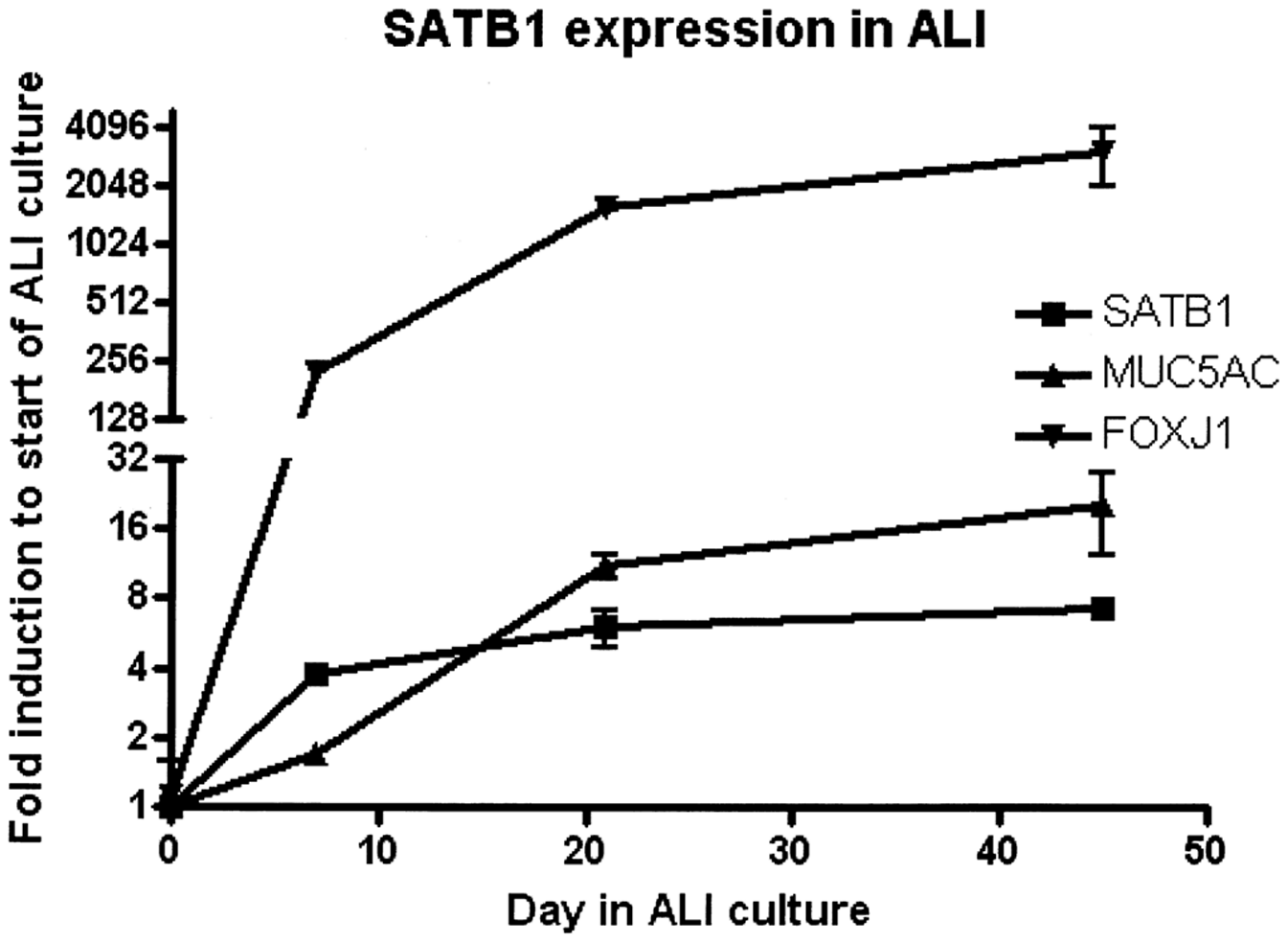
*SATB1*, *MUC5AC* and *FOXJ1* mRNA expression levels during mucociliary human airway epithelial cell differentiation (n = 2 donors). Expression of *SATB1*, the identified gene in our study, *MUC5AC* a marker of mucus, and *FOXJ1*, representing ciliated cells in epithelial cell culture on air liquid interface.

We investigated whether SATB1 expression was induced during mucociliary differentiation of primary human bronchial epithelial (HBE) cells *in vitro* and compared SATB1 mRNA expression levels at different time points of an air-liquid interface (ALI) culture for up to 45 days. ALI culture of HBE cells induced mucociliary differentiation, as confirmed by induction of expression of FOXJ1, a marker for ciliated cells (19) and MUC5AC, a marker of goblet cells. SATB1 expression was induced over time (Figure 6), with an approximately 8-fold increased expression from the start to the end of the 45-day ALI culture period.

## Discussion

Since not every ex- or current heavy smoker suffers from chronic mucus hypersecretion (CMH), we aimed to identify genetic variants conferring susceptibility to CMH. Therefore, we performed the first GWA study on CMH, the key presenting symptom in chronic bronchitis. CMH was associated with 36 SNPs at the p<10^−4^ significance level in the identification cohort. In the meta-analysis combining our identification and replication cohorts, strong association was observed with rs6577641, a SNP located on chromosome 3 in intron 9 of *SATB1*. Although the association of rs6577641 with CMH did not reach conventional genome-wide significance, its effect was in the same direction and was significant (4.25×10^−6^) at nominal levels (1.61×10^−3^) across eleven study populations, showing the robustness of this finding. The detected odds ratio for this SNP suggests an additional risk of 17% per G allele to develop CMH in a population of ex- and current heavy smokers.

Multivariate regression analysis, stratified for current an ex-smoking, showed essentially the same effect sizes and direction of the association of CMH and the risk allele of rs6577641. It is likely that lack of power is the reason for not reaching the level of significance in ex-smokers.

These data strongly suggest that *SATB1* plays a role in the susceptibility to CMH in subjects with a history of heavy smoking (≥20 pack-years) within the general population. Moreover, rs6577641 has a *cis*-eQTL effect on *SATB1* lung tissue expression, the risk allele at rs6577641 (G) increasing and the A-allele reducing expression of *SATB1* significantly. Additionally, we found a higher *SATB1* expression in bronchial biopsies of COPD-patients with CMH. We found no differences between the GG and AA genotypes for protein expression of SATB1 in airway epithelium by IHC in a small sample from our lung tissue registry. Finally, we demonstrate that *SATB1* mRNA expression is induced during mucociliary differentiation in ALI cultures of human bronchial epithelial cells of 2 donors supporting our eQTL findings. Interestingly, expression of the mucin gene *MUC5AC* was also induced during this culture period, with a slightly delayed kinetics compared to *SATB1*. Together these data strongly suggest that *SATB1* is induced during differentiation of bronchial epithelial cells and affects chronic mucus hypersecretion.

The forest plot clearly shows that the effect of SNP rs6577641 is lower in cohorts including COPD patients only (GLUCOLD, Rucphen, COPDGene, ECLIPSE and Norway) than in the other cohorts. Additional meta-analysis of COPD-cohorts and general population based cohorts separately confirmed this (COPD cohorts, combined p-value = 0.236, OR = 1.07 and general population based cohorts, combined p-value = 5.18×10^−7^, OR = 1.26). This suggests genetic heterogeneity of CMH in subjects with and without COPD.

The SNP most significantly associated with CMH, rs6577641, is located in an intron of *SATB1*. *SATB1* is a transcription factor and chromatin (re)organizer important for controlling the expression of many genes in a tissue or cell-type specific fashion, for instance in differentiating thymus T-cells [17] or differentiating skin keratinocytes [18]. Expression of SATB1 has been observed in normal human bronchial epithelial cells by immunohistochemistry and lower levels were observed in non-small lung cancer cells [16]. In our study, we also showed the presence of SATB1 in bronchial epithelial cells by IHC staining of lung tissue. However, no significant differences were found between patients homozygous for the protective and risk alleles, for either specific SATB1 staining or for PAS staining, the latter specifically detecting mucus. This inability to detect a genotype effect on protein staining may be due to lack of power, as we found a large variation in SATB1 and PAS protein expression in the relatively small number of lung tissue samples. Other explanations include possible expression regulation of SATB1 by smoke exposure which could be a dynamic process not readily detected at the protein level by any single-time point analysis such as IHC staining on lung biopsies. Alternatively SATB1 expression levels may vary throughout the lungs or the technique used here is not sensitive enough to detect relatively small differences in protein levels. To further explore the association of SATB1 protein and its underlying regulation, it would be of interest to perform longitudinal investigations on lung tissue samples of subjects with and without CMH, or time series of *in vitro* cultured epithelial cells from donors with a specific genotype and cigarette smoke exposure. This would also allow further studies on epigenetic regulation with methylation, microRNA or histone modifications.

The lack of association between the SATB1 protein and rs6577641 might additionally be due to the location of mucus positive cells in lung tissue. Mucus is produced both by goblet cells and submucosal glands, which we did not investigate further. Normal mucus consists of 97% water and 3% solids including 30% mucins. In case of dysregulation of mucus production, the concentration of solids in mucus may increase up to 15%. A further step therefore could involve investigating mucins/proteins present in mucus, e.g. MUC5AC is predominantly produced by goblet cells in proximal airways and MUC5B by secretory cells throughout the airways and by submucosal glands.

How does *SATB1* expression contribute to CMH? *SATB1* is known to be a genome organizer, a tissue specific chromatin remodeling protein with a property to modifying chromatin architecture by formation of loops, allowing contact of condensed genomic DNA to regulatory transcription proteins [19]. Thus *SATB1* can control gene expression of a series of target genes located within a single locus at a specific chromosomal location [20]. This has for instance been elegantly shown in case of differentiating keratinocytes [18], where Satb1 expression regulates genes located in the keratinocyte-specific loci, leading to adaptation of a specific cell fate of the differentiating keratinocytes. Similarly, a mechanism by which *SATB1* could contribute to CMH is the induction of a gene expression program during differentiation of bronchial epithelial cells, leading to adaptation of a cell fate specific for mucus producing cells in the submucosal glands or a goblet cell phenotype in the bronchial epithelium. Involvement of Satb1 in pneumocyte differentiation was previously observed by Baguma et al. in mice [21]. We observed induction of *SATB1* expression in bronchial epithelial cells differentiating under ALI culture conditions. Further research will need to test whether a specific gene expression profile is induced by *SATB1* expression in differentiating bronchial epithelial cells. SATB1 is also highly expressed in thymocytes, but absent in mature non-activated T cells [22]. Moreover, Satb1 has been shown in mice to be essential for expression of T_helper_2 (Th2) cells important in the regulation of genes encoding interleukin 4, 5 and 13 [19]. In Satb1-deficient mice, development of thymocytes stopped after the CD4^+^/CD8^+^ stage with deregulation of many genes [23]. Conversely, in case of excessive SATB1-production an excess of Th2 cells may be formed which all produce IL-13, which may contribute to increased mucus production. Therefore, a putative role of SATB1 in T-cells for the CMH phenotype should not be disregarded.

Strength of our study is the fact that we were able to replicate our findings in different populations, ranging from cohorts consisting of individuals with severe airflow limitation to cohorts mainly consisting of healthy smokers. There are some limitations, e.g. the presence of CMH was not based on actual measurements of the amount of sputum produced but based on questionnaires that were not completely similar in all study cohorts. Underreporting of CMH occurs since those experiencing CMH become accustomed to these symptoms, believing they are smoking related or because they are embarrassed to admit to cough and sputum. We demonstrated that *SATB1* mRNA expression is induced during mucociliary differentiation in ALI cultures of HBE cells in a small dataset (n = 2). However, these data seem reliable as they are supported by eQTL data from lung tissue. Despite this drawback, we consistently found evidence for association of *SATB1* with CMH in the populations studied, showing the robustness of our finding. Moreover, we corroborated this finding by functional studies in lung tissue, airway wall biopsies of COPD patients and epithelial cultures. More extensive research is needed to investigate which factors induce SATB1 expression in airway epithelium.

In summary, we performed identification analyses and meta-analyses using data from almost 7,000 participants to identify genes involved in susceptibility for CMH. It is remarkable that we found a genetic association for CMH given this phenotype is partly subjectively determined and not well delineated. Moreover, despite cohort differences to define CMH and severity of airflow limitation, we found consistent effects of SNP rs6577641 on CMH. This confirms that the CMH phenotype, despite the fact that it is self-reported, is a robust phenotype irrespective of the presence or absence of airflow limitation. The association of rs6577641 on chromosome 3 at the *SATB1* locus with CMH was supported by functional studies including gene expression findings, demonstrating SATB1 to be associated with CMH.

Chronic mucus hypersecretion is a bothersome symptom for many people, it increases in prevalence with aging and affects quality of life, exacerbations of symptoms due to respiratory infections and ultimately increases mortality. The involvement of *SATB1* in CMH offers opportunities to better understand the process leading to CMH, and future development of tailored medicines.

## Supporting Information

Supplement S1 (DOC)
