# Correction: Susceptibility to Chronic Mucus Hypersecretion, a Genome Wide Association Study

**DOI:** 10.1371/journal.pone.0129524

**Published:** 2015-05-29

**Authors:** Akkelies E. Dijkstra, Joanna Smolonska, Maarten van den Berge, Ciska Wijmenga, Pieter Zanen, Marjan A. Luinge, Mathieu Platteel, Jan-Willem Lammers, Magnus Dahlback, Kerrie Tosh, Pieter S. Hiemstra, Peter J. Sterk, Avi Spira, Jorgen Vestbo, Borge G. Nordestgaard, Marianne Benn, Sune F. Nielsen, Morten Dahl, W. Monique Verschuren, H. Susan J. Picavet, Henriette A. Smit, Michael Owsijewitsch, Hans U. Kauczor, Harry J. de Koning, Eva Nizankowska-Mogilnicka, Filip Mejza, Pawel Nastalek, Cleo C. van Diemen, Michael H. Cho, Edwin K. Silverman, James D. Crapo, Terri H. Beaty, David A. Lomas, Per Bakke, Amund Gulsvik, Yohan Bossé, Ma’en Obeidat, Daan W. Loth, Lies Lahousse, Fernando Rivadeneira, Andre G. Uitterlinden, Andre Hofman, Bruno H. Stricker, Guy G. Brusselle, Cornelia M. van Duijn, Uilke Brouwer, Gerard H. Koppelman, Judith M. Vonk, Martijn C. Nawijn, Harry J. M. Groen, Wim Timens, H. Marike Boezen, Dirkje S. Postma, the LifeLines Cohort study

The 37^th^ author’s name is spelled incorrectly. The correct name is: Ma’en Obeidat.
