# Supplementary material for: Susceptibility to Chronic Mucus Hypersecretion, a Genome Wide Association Study
Source: PLoS One. 2014 Apr 8;9(4):e91621. doi: 10.1371/journal.pone.0091621 (PMC3979657; doi:10.1371/journal.pone.0091621)
Supplement: Supplement S1 — (DOC) [file pone.0091621.s001.doc]

Supporting information according to:

Susceptibility to chronic mucus hypersecretion,

a genome wide association study

**Populations, genotyping and quality control**

The *NELSON-study* is a Dutch multi-center lung cancer screening study and includes only male current or former smokers (≥ 20 pack-years). Detailed inclusion criteria and characteristics have been described elsewhere [1].

Genome-wide single nucleotide polymorphism (SNP) genotyping for NELSON individuals was performed on Illumina Human 610-Quad BeadChip containing over 620,000 markers. Genotypes were called with the standard algorithm provided by Illumina and implemented in Genome Studio software. Quality control (QC) in NELSON implied exclusion of those individuals from whom > 5% of genotyping was missing, when detected as an ethnic outlier or when a sample was derived from a relative of another participant (based on genetic distance derived from principal components c1 and c2, and on IBS estimation, Phat > 0.5) and when lung cancer was present. SNPs were excluded if minor allele frequency (MAF) was < 5%, if a deviation from Hardy-Weinberg was observed (p < 0.0001) and if > 5% of samples were missing.

In the first GWA on CMH (with adjustment for center only), we included blood bank controls to increase the power. However, except for gender, there was no information available for these controls. We could not include these controls in the second GWA analysis (with adjustment for center and ex-/current smoking) as we had no information on smoking habits.

*COPACETIC-populations*

*The Doetinchem Study* is a general population cohort of the inhabitants of Doetinchem, an industrial town in the Netherlands. We used data collected between 1998 and 2002. Detailed inclusion criteria and characteristics have been described elsewhere [2].

*The Vlagtwedde-Vlaardingen Study* is a Dutch population based study based on a random sample of the general population from Vlagtwedde (a rural village) and Vlaardingen (an industrial village). We used data collected between 1989 and 1990. Detailed inclusion criteria and characteristics have been described elsewhere [3,4].

*GLUCOLD* is an acronym for Groningen Leiden Universities and Corticosteroids in Obstructive Lung Disease. The GLUCOLD Study is a multicenter trial including COPD-patients. All patients had a history of ≥ 10 pack-years smoking and COPD-stage II or higher. Detailed inclusion criteria and characteristics have been described previously [5].

*Poland* is a general population based Polish cohort participating in the Burden of Lung Disease (BOLD) initiative, an international study designed to investigate COPD prevalence and economic burden. The Polish population consisted of a random sample aged ≥ 40 years from the Malopolska region in southern Poland. Detailed inclusion criteria and characteristics have been described elsewhere [6,7].

Participants in the *Rucphen Study* are part of a genetically isolated population located in the southwest of The Netherlands. All participants were heavy smokers and had COPD stage II or higher based on GOLD-guidelines [4].

The *Heidelberg* cohort is a German cohort participating in a Lung Cancer Screening Intervention Trial using the same inclusion criteria as the NELSON study [8].

Genotyping of top-SNPs for replication in COPACETIC-cohorts was performed on custom made Veracode assays. QC in COPACETIC-cohorts implied exclusion of those individuals from whom > 10% of genotyping was missing. SNPs were excluded if minor allele frequency (MAF) was < 5%, if a deviation from Hardy-Weinberg was observed (p < 0.0001) and if > 5% of samples were missing.

*Non COPACETIC populations*

The *Rotterdam Study* is a prospective population-based cohort study founded in 1990 in a suburb of Rotterdam, the Netherlands. The first cohort (RS I) consists of 7,983 participants, aged 55 years and over. The second cohort (RS II) was recruited in 2000 with the same inclusion criteria. The third cohort (RS III) was recruited in 2006. Details regarding the Rotterdam study have been described elsewhere [9,10].

The *LifeLines Cohort Study* is a prospective population based cohort study on multimorbidity, being conducted in the northern part of the Netherlands [11].

*Norway* is a COPD (GOLD stage II or worse) case-control cohort from Bergen, Norway. Details regarding the NORWAY study have been published previously [12,13]. All participants were ex- or current smokers with ≥ 2.5 pack-years and age ≥ 40 years.

*COPDGene* is a multicenter study including subjects from 21 clinical study centers throughout USA with: a smoking history of ≥ 10 pack-years. Subjects with COPD (GOLD II-IV) and normal spirometry were included. Only non-Hispanic White subjects from COPDGene were included in this analysis. Details regarding the COPDGene study have been described elsewhere [14].

*ECLIPSE*, the Evaluation of COPD Longitudinally to Identify Predictive Surrogate Endpoints, is a multicenter case-control study including individuals from 46 centers in 12 countries. Details regarding the ECLIPSE study have been published previously [15]. COPD-cases (GOLD II–IV, ≥ 10 pack-years), smoking controls (≥10 pack-years) and non smoking controls (< 1 pack-year) without COPD are included. Only Caucasian subjects were included in this analysis.

The non-COPACETIC-studies performed their own genotyping using different commercially available platforms, quality control and imputation. Imputations of non-genotyped SNPs were carried out within each study.

*Ethics statement (detailed information)*

The trial was approved by the Dutch Ministry of Health and the ethics board at

each Dutch participating center. The names of the approving institutional

review boards or equivalent committee(s) of all centers are listed here below.

Groningen: The Medical Ethics Committee of the University Medical Center Groningen & the Medical Ethics Committee of the University Medical Center Utrecht

LifeLines: The Medical Ethics Committee of the University Medical Center Groningen

GLUCOLD: The Medical Ethics Committee of the University Medical Center Groningen & the Medical Ethics Committee of the Leiden University Medical Center

Utrecht: The Medical Ethics Committee of the University Medical Center Utrecht

Rotterdam: The Medical Ethics Committee of the Erasmus Medical Center Rotterdam

Rucphen: The Medical Ethics Committee of the Erasmus Medical Center Rotterdam

Vlagtwedde- The Medical Ethics Committee of the University

Vlaardingen: Medical Center Groningen

Doetinchem: The Medical Ethics Committee of the Netherlands Organization for Applied Scientific Research (TNO)

Poland: The Bioethics Committee of the Jagiellonian University Medical School, Krakow

Heidelberg: The ethical committee of the medical faculty of the University of Heidelberg

Norway: The Regional Ethics Committee in Western Norway

ECLIPSE: The ECLIPSE study was approved by the institutional review boards of all participating centers. The ethics committees/institutional review boards of the participating centers are listed here below

| **Institutions** | **City** | **Country** |
| --- | --- | --- |
| University of Texas Health Science Center | San Antonio | USA |
| Rhode Island Hospital | Providence | USA |
| Los Angeles Biomedical Research Institute at Harbor-UCLA Medical Center | Torrance | USA |
| St. Elizabeth's Medical Center | Boston | USA |
| Pulmonary Associates of Richmond, Inc. | Richmond | USA |
| Pulmonary Associates, PA | Phoenix | USA |
| Advances in Medicine | Rancho Mirage | USA |
| Baylor Clinic | Houston | USA |
| Dartmouth-Hitchcock Medical Center | Lebanon | USA |
| National Jewish Medical & Research Center | Denver | USA |
| University of Nebraska Medical Center | Omaha | USA |
| Yale University School of Medicine | New Haven | USA |
| Mayo Clinic | Rochester | USA |
| Creighton University Medical Center | Omaha | USA |
| University of Pittsburgh Medical Center Emphysema Research Center | Pittsburgh | USA |
| Houston VA Medical Center | Houston | USA |
| Midwest Chest Consultants, PC | St. Charles | USA |
| Harvard University-Brigham & Women's Hospital | Boston | USA |
| University of Miami School of Medicine | Miami | USA |
| Johns Hopkins Asthma & Allergy Center | Baltimore | USA |
| St. Francis Hospital & Medical Center | Hartford | USA |
| Montreal Chest Institute | Montreal | Canada |
| UBC - Respiratory Medicine | Vancouver | Canada |
| Queen Elizabeth II Health Sciences Center | Halifax | Canada |
| McMaster University, Health Sciences Center | Hamilton | Canada |
| Pacific Lung Health Center | Vancouver | Canada |
| Centre de recherche Hospital Laval | Sainte-Foy | Canada |
| Kingston General Hospital | Kingston | Canada |
| Institut Clínic del Tòrax | Barcelona | Spain |
| HAUKELAND UNIVERSITY HOSPITAL | Bergen | Norway |
| Aintree University Hospital | Liverpool | UK |
| Asthma Center | Pleven | [Bulgaria](../../../../../AppData/Local/Microsoft/AppData/Documents%20and%20Settings/rac40917/Local%20Settings/Temp/Documents%20and%20Settings/SVT61472/Local%20Settings/Temp/Local%20Settings/Temp/Local%20Settings/Temp/Local%20Settings/Temp/Local%20Settings/alg11089/Desktop/center_detail.asp) |
| KOPA Golnik | Golnik | Slovenia |
| Military Medical Academy, Department of Pulmonology | Sofia | Bulgaria |
| SPliN s.r.o | Praha | Czech Republic |
| Cambridge Institute of Medical Research | Cambridge | UK |
| New Royal Infirmary Of Edinburgh | Edinburgh | UK |
| P3 Research | Wellington | NZ |
| Medicines Evaluation Unit - Langley Bldg. | Manchester | UK |
| H:S Hvidovre Hospital | Hvidovre | Denmark |
| The Royal Free Hospital | London | UK |
| Astmacentrum Hornerheide | Horn | Netherlands |
| Institute of Phthisiatry & Pulmonology | Kiev | Ukraine |
| Institute of Phthisiatry & Pulmonology | Kiev | Ukraine |
| Institute of Phthisiatry & Pulmonology | Kiev | Ukraine |
| Donetsk State Medical University | Donetsk | Ukraine |

COPDGene: The COPDGene study was approved by the institutional review boards of all participating COPDGene centers. The participating centers are listed below.

| **Institutions** | **City** | **Country** |
| --- | --- | --- |
| Ann Arbor VA | Ann Arbor | USA |
| Baylor College of Medicine | Houston | USA |
| Brigham and Women's Hospital | Boston | USA |
| Columbia University | New York | USA |
| Duke University Medical Center | Durham | USA |
| Fallon Clinic | Worcester | USA |
| Health Partners Research Foundation | Minneapolis | USA |
| Johns Hopkins University | Baltimore | USA |
| Los Angeles Biomedical Research Institute at Harbor UCLA Medical Center | Los Angeles | USA |
| Michael E. DeBakey VAMC | Houston | USA |
| Minneapolis VA | Minneapolis | USA |
| Morehouse School of Medicine | Atlanta | USA |
| National Jewish Health | Denver | USA |
| Temple University | Philadelphia | USA |
| University of Alabama | Birmingham | USA |
| University of California | San Diego | USA |
| University of Iowa | Iowa City | USA |
| University of Michigan | Ann Arbor | USA |
| University of Minnesota | Minneapolis | USA |
| University of Pittsburgh | Pittsburgh | USA |
| University of Texas Health Science Center at San Antonio | San Antonio | USA |

**The lung eQTL study**

Non-tumor lung tissues were collected from patients who underwent lung resection surgery at three participating sites: Laval University (Quebec City, Canada), University of Groningen (Groningen, The Netherlands), and University of British Columbia (Vancouver, Canada). Whole-genome gene expression and genotyping data were obtained from these specimens. Gene expression profiling was performed using an Affymetrix custom array testing 51,627 non-control probe sets and normalized using RMA (Irizarry, R.A. et al. Biostatistics 2003; 4, 249-64). Genotyping was performed using the Illumina Human1M-Duo BeadChip array. At Laval, lung specimens were collected from patients undergoing lung cancer surgery and stored at the “Institut universitaire de cardiologie et de pneumologie de Québec” (IUCPQ) site of the Respiratory Health Network Tissue Bank of the “Fonds de recherche du Québec – Santé” (www.tissuebank.ca). Written informed consent was obtained from all subjects and the study was approved by the IUCPQ ethics committee. At Groningen, lung specimens were provided by the local tissue bank of the Department of Pathology and the study protocol was consistent with the Research Code of the University Medical Center Groningen and Dutch national ethical and professional guidelines (“Code of conduct; Dutch federation of biomedical scientific societies”; http://www.federa.org). At Vancouver, the lung specimens were provided by the James Hogg Research Center Biobank at St Paul's Hospital and subjects provided written informed consent. The study was approved by the ethics committees at the UBC-Providence Health Care Research Institute Ethics Board.

The lung eQTL analysis was performed as described before by Fehrman and Hao [16,17].

**Gene expression analysis in GLUCOLD**

*RNA Isolation and Size Fractionation*

Out of 114 COPD subjects in GLUCOLD, 89 individuals had endobronchial biopsies which had been immediately snap-frozen, stored at -80 ^o^C and were available for extraction of RNA. RNA was extracted from bronchial biopsies and fractioned into low molecular weight (<200 nt) and high molecular weight (>200 nt) fractions using the miRNeasy mini kit (QIAGEN) according to manufacturer’s protocol. The purity of RNA fractions was assessed using a NanoDrop 1000 UV-Vis spectrophotometer, and the integrity of the large RNA fraction was assessed by using the RNA Pico assay in the Agilent 2100 BioAnalyzer.

*RNA processing and microarray hybridization*

All procedures were performed at Boston University Microarray Resource Facility as described in GeneChip® Whole Transcript (WT) Sense Target Labeling Assay Manual (Affymetrix, Santa Clara, CA, current version available at www.affymetrix.com). The Qiagen miRNeasy Mini Kit and RNeasy MinElute Cleanup Kit were used to isolate high and low molecular weight RNA. 200 ng of high molecular weight large RNA was reverse transcribed using the Whole Transcript cDNA Synthesis kit (Affymetrix, Santa Clara, CA). The obtained cDNA was used as a template for in vitro transcription using the Whole Transcript cDNA Amplification Kit (Affymetrix, Santa Clara, CA). The obtained antisense cRNA was purified using GeneChip Sample Cleanup Module (Affymetrix, Santa Clara, CA), and used as a template for reverse transcription (Whole Transcript cDNA Synthesis kit, Affymetrix, Santa Clara, CA) to produce single-stranded DNA in the sense orientation. During this step, dUTP was incorporated. The DNA was then fragmented using uracil DNA glycosylase (UDG) and apurinic/apyrimidinic endonuclease 1 (APE 1) and labeled with DNA Labeling Reagent that was covalently linked to biotin using terminal deoxynucleotidyl transferase (TdT, Whole Transcript Terminal Labeling kit, Affymetrix, Santa Clara, CA). IVT and cDNA fragmentation quality controls were carried out by running an mRNA Nano assay in the Agilent 2100 Bioanalyzer. The labeled fragmented DNA was hybridized to the Affymetrix Human Gene 1.0 ST Arrays for 16-18 hours in GeneChip Hybridization oven 640 at 45oC with rotation (60 rpm). The hybridized samples were washed and stained using Affymetrix fluidics station 450. The first stain with streptavidin-R-phycoerythrin (SAPE) was followed by signal amplification using a biotinilated goat anti-streptavidin antibody and another SAPE staining (Hybridization, Washing and Staining Kit, Affymetrix, Santa Clara, CA). Microarrays were immediately scanned using Affymetrix GeneArray Scanner 3000 7G Plus (Affymetrix, Santa Clara, CA).

*Data acquisition, probeset summarization and normalization, and data preprocessing*

Normalization was performed with Affymetrix Expression Console software using Affymetrix default Robust Multichip Analysis (RMA) sketch algorithm workflow and 1 additional sample was excluded due to low quality of the microarray data.

Microarray data quality was assessed using relative log expression (RLE) plots, normalized unscaled standard error (NUSE) plots, and principle component analysis (PCA). Based on the RLE and NUSE plots, a total of 9 microarrays were excluded, leaving 79 microarrays for subsequent analysis, 77 having data on CMH (38 CMH-cases, 39 non-CMH-controls).

Association of CMH with *SATB1* mRNA-expression levels was analyzed with logistic regression and adjustment for current smoking and RNA integrity score.

**Gene expression levels during airway epithelial cell differentiation**

To investigate *SATB1* gene expression levels during airway epithelial cell differentiation, a time-course series of air liquid interface cultured cells (ALIs) and submerged cultured cells was purchased (MucilAirTM, Epithelix Sàrl, Geneva, Switzerland) and analyzed as described before [18]. ALI cultured cells were analyzed harvested from two independent culture series (duplicate), whereas submerged cultured cells were analyzed from a single series of cultures. Briefly, at time-points 1, 7, 21 and 45 days after start of ALI culture, cells were harvested for RNA and analyzed for gene expression levels by qRT-PCR analysis using Taqman Assays (Applied Biosystems Europe BV, Nieuwekerk A/D IJssel, the Netherlands). qRT-PCRs were performed on the ABI7900HT cycler in 384-well format. Pre-designed assays were used for the detection of *SATB1* (Hs00161515_m1), FOXJ1 (Hs00230964_m1), MUC5AC (Hs01365616_m1) and four house-keeping genes: GAPDH (Hs99999905_m1), β-actin (Hs99999903_m1), and RPLPO (Hs99999902_m1). cDNA was produced using OmniscriptTM Reverse Transcriptase (QIAGEN Benelux BV, Venlo, the Netherlands). A total of 500 ng of RNA was reverse transcribed into cDNA using Oligo-12-18) primers in a 20 μl reaction volume, including RNase inhibitor, at 37oC for 1 hour. Each qPCR reaction contained 17.5ng of cDNA, 250nM of probe, 900nM of forward and reverse primers, 5 μl TaqMan Universal PCR Master Mix (Applied Biosystems Europe BV, Nieuwekerk A/D IJssel, The Netherlands), in a final volume of 10 μl. All samples were measured in duplicate using recommended cycling conditions. Data was analyzed using SDS2.3 software by applying the ΔΔCt-method (Applied Biosystems User Bulletin 2). As three house-keeping genes were used, the best combination of house-keeping genes for normalization was determined by using the Normfinder applet.

**Immunohistochemical staining**

Bronchial tissue for SATB1- and PAS staining was available from patients in whom lung surgery or lung transplantation was conducted because of COPD or lung cancer.

*SATB1-staining*

Monoclonal mouse anti-SATB1 (BD cat nr. 611182) was used for immunohistochemistry. Antigen retrieval was performed with 10mM Tris/1mMEDTA buffer pH 9.0 at 125 ̊C for 15 minutes in a Pascal pressure chamber (Dako), and the primary antibody was incubated overnight at 4 ̊C in a 1/100 dilution. The incubation of the second step, rabbit anti mouse 1/100, and the third step, goat anti rabbit 1/100, was 30 minutes. The color reaction was with Di-Amino Benzidin (DAB; Sigma, Illinois). Tonsil tissue was used as a positive control.

The number of strong positively-, positively- and weak positively stained cells per case was counted in the epithelium. Quantifiable areas were selected for evaluation when the following requirements were met 1) SATB1 and HE staining was good 2) intact basement membrane (BM) 3) presence of maximal 1-3 layer(s) of epithelial cells, including a layer of ciliated epithelium, thus avoiding hyperplasia and squamous metaplasia.

*PAS-staining*

Periodic acid-shift (PAS) histochemical staining was performed using the DAKO autostainer (DAKO, Glostrup, Denmark) at the Pathology department.

Areas were selected for evaluation when the following requirements were met 1) PAS staining was acceptable 2) intact basement membrane (BM) 3) presence of maximal 1-3 layer(s) of epithelial cells, including a layer of ciliated epithelium.

The number of PAS-positive pixels was determined in the epithelium and expressed as the percentage of mucus-positivity in all measured epithelium per sample by using Aperio® Scanscope software.

All stainings were quantiﬁed by a blinded observer.

References

1. van Klaveren RJ, Oudkerk M, Prokop M, Scholten ET, Nackaerts K, et al. (2009) Management of lung nodules detected by volume CT scanning. N Engl J Med 361: 2221-2229.

2. Verschuren WM, Blokstra A, Picavet HS, Smit HA. (2008) Cohort profile: The doetinchem cohort study. Int J Epidemiol 37: 1236-1241.

3. Rijcken B, Schouten JP, Mensinga TT, Weiss ST, De Vries K, et al. (1993) Factors associated with bronchial responsiveness to histamine in a population sample of adults. Am Rev Respir Dis 147: 1447-1453.

4. van Diemen CC, Postma DS, Vonk JM, Bruinenberg M, Nolte IM, et al. (2006) Decorin and TGF-beta1 polymorphisms and development of COPD in a general population. Respir Res 7: 89.

5. Lapperre TS, Snoeck-Stroband JB, Gosman MM, Jansen DF, van Schadewijk A, et al. (2009) Effect of fluticasone with and without salmeterol on pulmonary outcomes in chronic obstructive pulmonary disease: A randomized trial. Ann Intern Med 151: 517-527.

6. Nizankowska-Mogilnicka E, Mejza F, Buist AS, Vollmer WM, Skucha W, et al. (2007) Prevalence of COPD and tobacco smoking in malopolska region--results from the BOLD study in poland. Pol Arch Med Wewn 117: 402-410.

7. Lamprecht B, McBurnie MA, Vollmer WM, Gudmundsson G, Welte T, et al. (2011) COPD in never smokers: Results from the population-based burden of obstructive lung disease study. Chest 139: 752-763.

8. Becker N, Delorme S, Kauczor H,U. (2008) LUSI: The german component of the european trial on the efficacy of multislice-CT for the early detection of lung cancer. onkologie 31 .

9. Soler Artigas M, Loth DW, Wain LV, Gharib SA, Obeidat M, et al. (2011) Genome-wide association and large-scale follow up identifies 16 new loci influencing lung function. Nat Genet 43: 1082-1090.

10. Hofman A, van Duijn CM, Franco OH, Ikram MA, Janssen HL, et al. (2011) The rotterdam study: 2012 objectives and design update. Eur J Epidemiol 26: 657-686.

11. Stolk RP, Rosmalen JG, Postma DS, de Boer RA, Navis G, et al. (2008) Universal risk factors for multifactorial diseases: LifeLines: A three-generation population-based study. Eur J Epidemiol 23: 67-74.

12. Cho MH, Castaldi PJ, Wan ES, Siedlinski M, Hersh CP, et al. (2011) A genome-wide association study of COPD identifies a susceptibility locus on chromosome 19q13. Hum Mol Genet .

13. Grydeland TB, Dirksen A, Coxson HO, Pillai SG, Sharma S, et al. (2009) Quantitative computed tomography: Emphysema and airway wall thickness by sex, age and smoking. Eur Respir J 34: 858-865.

14. Regan EA, Hokanson JE, Murphy JR, Make B, Lynch DA, et al. (2010) Genetic epidemiology of COPD (COPDGene) study design. COPD 7: 32-43.

15. Vestbo J, Anderson W, Coxson HO, Crim C, Dawber F, et al. (2008) Evaluation of COPD longitudinally to identify predictive surrogate end-points (ECLIPSE). Eur Respir J 31: 869-873.

16. Fehrmann RS, Jansen RC, Veldink JH, Westra HJ, Arends D, et al. (2011) Trans-eQTLs reveal that independent genetic variants associated with a complex phenotype converge on intermediate genes, with a major role for the HLA. PLoS Genet 7: e1002197.

17. Hao K, Bosse Y, Nickle DC, Pare PD, Postma DS, et al. (2012) Lung eQTLs to help reveal the molecular underpinnings of asthma. PLoS Genet 8: e1003029.

18. Koning H, Sayers I, Stewart CE, de Jong D, Ten Hacken NH, et al. (2012) Characterization of protocadherin-1 expression in primary bronchial epithelial cells: Association with epithelial cell differentiation. FASEB J 26: 439-448.
